# Polyhydroxyalkanoate Synthesis by *Burkholderia glumae* into a Sustainable Sugarcane Biorefinery Concept

**DOI:** 10.3389/fbioe.2020.631284

**Published:** 2021-01-13

**Authors:** Carolina Bilia Chimello de Paula, Fabrício Coutinho de Paula-Elias, Marcela Nogueira Rodrigues, Luciana Fontes Coelho, Nayra Morgana Lima de Oliveira, Alex Fernando de Almeida, Jonas Contiero

**Affiliations:** ^1^Institute for Research in Bioenergy, São Paulo State University, Rio Claro, Brazil; ^2^Graduate Program on Food Science and Technology, Federal University of Tocantins, Palmas, Brazil; ^3^Institute of Biosciences, São Paulo State University, Rio Claro, Brazil; ^4^Graduate Program on Food Science and Technology, Federal University of Tocantins, Gurupi, Brazil

**Keywords:** bioplastic, byproduct, molasses, vinasse, bagasse

## Abstract

Polyhydroxyalkanoate (PHA) bioplastic was synthesized by *Burkholderia glumae* MA13 from carbon sources and industrial byproducts related to sugarcane biorefineries: sucrose, xylose, molasses, vinasse, bagasse hydrolysate, yeast extract, yeast autolysate, and inactivated dry yeast besides different inorganic nitrogen sources. Sugarcane molasses free of pre-treatment was the best carbon source, even compared to pure sucrose, with intracellular polymer accumulation values of 41.1–46.6% cell dry weight. Whereas, xylose and bagasse hydrolysate were poor inducers of microbial growth and polymer synthesis, the addition of 25% (v/v) sugarcane vinasse to the culture media containing molasses was not deleterious and resulted in a statistically similar maximum polymer content of 44.8% and a maximum PHA yield of 0.18 g/g, at 34°C and initial pH of 6.5, which is economic and ecologically interesting to save water required for the industrial processes and especially to offer a fermentative recycling for this final byproduct from bioethanol industry, as an alternative to its inappropriate disposal in water bodies and soil contamination. Ammonium sulfate was better even than tested organic nitrogen sources to trigger the PHA synthesis with polymer content ranging from 29.7 to 44.8%. GC-MS analysis showed a biopolymer constituted mainly of poly(3-hydroxybutyrate) although low fractions of 3-hydroxyvalerate monomer were achieved, which were not higher than 1.5 mol% free of copolymer precursors. *B. glumae* MA13 has been demonstrated to be adapted to synthesize bioplastics from different sugarcane feedstocks and corroborates to support a biorefinery concept with value-added green chemicals for the sugarcane productive chain with additional ecologic benefits into a sustainable model.

## Introduction

Polyhydroxyalkanoates (PHAs) are microbial polyesters synthesized by representatives of Bacteria and Archaea domains as intracellular granules. PHAs are mainly associated to energy storage under culture conditions of carbon excess and nutrient imbalance such as nitrogen limitation. On the other hand, PHA granules have been also involved in microbial stress resistance with additional robustness and fitness of prokaryote cells (Steinbüchel and Füchtenbusch, 1998; Obruca et al., 2018; Koller, 2019). Beyond microbial adaptation strategies, PHAs are a family of bioplastics which are fully bio-based and biodegradable. These both characteristics are especially interesting since the term “bioplastics” have commonly been used to make a distinction from petrochemical and non-environmentally friendly polymers, which is partially misleading. Since bioplastics fulfill at least bio-based or biodegradable properties, there are many “drop-in” solutions such as bio-PP (bio-polypropylene) and bio-PET (bio-polyethylene terephthalate) that are partially or fully bio-based and a renewable alternative to petroleum-based polymers. However, these “drop-in” bioplastics are identical to their petrochemical counterparts and so they are not biodegradable and take many decades do be decomposed in nature. PHAs are not only bio-based and biodegradable but they also are compostable resulting in humus-rich soil (Chanprateep, 2010; Lackner, 2015; de Paula et al., 2018a; European Bioplastics, 2018). Furthermore, these microbial biopolymers are biocompatible, which is attractive for pharmaceutical and medical applications such as nanobeads for drug delivery systems and nanofiber scaffolds for tissue engineering (Rodriguez-Contreras, 2019). Additionally, PHAs meet the standard specification for marine degradability. All these mentioned features make PHAs promising candidates for short-lived and disposable applications (de Paula et al., 2018b).

The production of bioplastics has become more professional and differentiated in recent years with bio-based alternatives for practically every application. The bioplastic market has continually grown supported by climate goals and global awareness about sustainable outlets to the current energy sources with their capacities and production rates expected to maintain a CAGR (Compound Annual Growth Rate) of about 3% until 2024, which is almost the same of petrochemical plastics and fuels. Although the bioplastic market has drawn the attention of many industrial sectors, the market share of bio-based polymers is still modest and it is 1% of total polymer market (3.8 million tons in 2019). The main drivers of bioplastic industry have been poly(butylene adipate-*co*-terephthalate) (PBAT), epoxy resins, starch-containing polymers, polybutylene succinate (PBS), besides “drop-in” solutions such as bio-PP, which was first time available in 2019. Among these promising bioplastics, PHA capacities are expected to increase significantly by 2024 and many investors are betting on their dynamic market development (Carus, 2020). The cost effectiveness is the main challenge for a broad application of PHAs. For this reason, several scientists and companies have focused their efforts on technological upgrades with new polymer extraction methods and cost reduction based on fermentation strategies using microbial strains adapted to industrial byproducts (de Paula et al., 2018b). A biorefinery concept for biofuels and bioplastics has been proposed by many authors as an associated production set in order to generate value-added chemicals, especially those obtained from biofuel related byproducts, in order to support such industries as a production set partially or totally independent of petrochemical derivatives (Ashby et al., 2011; Cavalheiro et al., 2012; de Paula et al., 2017, 2019a).

Bioethanol is the largest produced transportation biofuel of the world with a global production projected to increase from about 122 bln L in 2019 to 143 bln L by 2028, which has been supported especially by blending mandates (OECD/FAO, 2019). The USA is the biggest bioethanol producer which is mainly obtained by corn starch fermentation with a production of 63.8 bln L in 2019 (U.S. Energy Information Administration, 2020), whereas Brazil is the second largest producer with a 2019/20 sugarcane harvest generating 35.7 bln L of bioethanol (CONAB, 2020). In the sugarcane biorefinery model is obtained about 11 tons of sugar and 7,000 L of bioethanol per hectare of processed sugarcane. From these processes are generated a diversity of byproducts such as sugarcane bagasse, straw, molasses, vinasse, and yeasts. Lignocellulosic biomass (sugarcane bagasse and straw) accounts two-thirds of sugarcane production. Molasses is a high sugar content byproduct which is obtained at the rate of 40 kg/ton of sugarcane. The bioethanol production is based on the fermentation of sugarcane juice or mixtures of broth and molasses by the yeast *Saccharomyces cerevisiae* achieving a concentration of 7–10% (v/v) ethanol after 6–10 h, when its own metabolite becomes toxic to the yeast and compromises the process viability (Santos et al., 2020). Yeast cells are collected at the end of fermentation cycle and a liquid-rich byproduct is obtained, sugarcane vinasse, which is an acid suspension with high COD values and accounts 10–15 L for each liter of produced ethanol (Christofoletti et al., 2013; Reis and Hu, 2017).

Vinasse has been mostly used on fertirrigation practices. However, the long term utilization of vinasse as liquid fertilizer implies negative effects on soil and ground water (Rocha et al., 2007; Christofoletti et al., 2013; Reis and Hu, 2017). Despite its low concentration of nitrogen and phosphorus, sugarcane vinasse has been used as nutrient source for microbial cultivations, which may serve as a treatment of vinasse reducing COD and toxic compounds besides to recycle the water back to the fermentation process and thus reducing the expenses with water and effluent treatment (Reis and Hu, 2017). Additionally, microbial fermentation of sugarcane vinasse is not only attractive for bioremediation purposes but also as a source of microbial metabolites with value-added such as bioplastics. In this sense, *Burkholderia glumae* MA13 was previously isolated by our scientific group and demonstrated to be a promising bacterial strain for PHA production from biodiesel byproducts (de Paula et al., 2019b). In this work, *B. glumae* MA13 was tested for biopolymer synthesis into a sugarcane biorefinery concept using sucrose, xylose, sugarcane molasses, vinasse, and bagasse hydrolysate in order to state the capabilities of this wild bacterial strain to produce bioplastics into a broad set of biomass-to-product process chain involving biofuels.

## Materials and Methods

### Microorganism and Culture Media

*Burkholderia glumae* MA13, previously isolated from Atlantic rainforest in Ubatuba, São Paulo State, Brazil, was used for bacterial cultivations and PHA synthesis (de Paula et al., 2019b). Lisogeny broth (LB: 10 g/L tryptone, 5 g/L yeast extract and 5 g/L NaCl) was utilized for inoculum preparation and bacterial maintenance in Petri plates and ultra-low temperature freezer at −80°C, stored in cryovials containing a final glycerol concentration of 20% (v/v). The culture medium pH was adjusted to all tested initial pH values (5.5–7.0) using 1 M NaOH or 1 M H_2_SO_4_. The basal medium for PHA production was mineral salts medium (MSM) (Ramsay et al., 1990) containing (g/L): Na_2_HPO_4_, 3.5; KH_2_PO_4_, 1.5; (NH_4_)_2_SO_4_, 1.0; MgSO_4_.7H_2_O, 0.2; CaCl_2_.2H_2_O, 0.01; Fe(III)NH_4_-citrate, 0.06; and 1 mL trace elements solution containing (g/L): H_3_BO_3_, 0.3; CoCl_2_.6H_2_O, 0.2; ZnSO_4_.7H_2_O, 0.1; MnCl_2_.4H_2_O, 0.03; NaMoO_4_ 2H_2_O, 0.03; NiCl_2_·6H_2_O, 0.02; CuSO_4_.5H_2_O, 0,01. Pure carbon sources and industrial byproducts related to sugarcane biorefineries were added to MSM as PHA precursors. Arabinose, Fructose, Glucose, Glycerol, Sucrose, and Xylose were tested as pure carbon sources. Sugarcane molasses was provided by São Domingos sugarcane biorefinery (Catanduva, Brazil) and it was added to culture media free of pre-treatment. Sugarcane vinasse was added to the media at different volume ratios (25, 50, and 100% v/v). This bioethanol refinery byproduct was centrifuged at 10,000 *g* for 10 min to remove remaining solid debris and yeast cells. Sugarcane bagasse was treated following 4 steps to generate bagasse hydrolysate. Firstly, 30 g sugarcane bagasse was added to 300 mL H_2_SO_4_ solution ranging from 0.5 to 5% (v/v) at 121 °C and 1 atm for 30 min. The liquid phase was separated by vacuum filtration using filter paper. In the second step, the filtered solution was submitted to overliming due to addition of anhydrous Ca(OH)_2_ up to the pH 10 and thereafter reduced to pH 7 by addition of 5 M H_2_SO_4_. The liquid phase was separated from precipitated gypsum by vacuum filtration. The third treatment step of sugarcane bagasse hydrolysate was initialized by heating the resulting solution at 80°C for 30 min under vacuum system in a rotary evaporator. Finally, in the fourth step, the concentrated bagasse hydrolysate was treated with 20% (w/v) activated charcoal for 3 h at 30°C in a rotary shaker at 150 rpm. Both sugarcane bagasse and vinasse were provided by Continental sugarcane biorefinery (Colômbia, Brazil). Crude glycerol from Petrobras biodiesel plant (Candeias, Brazil) was also utilized as a control byproduct in order to compare the bacterial performance for polymer synthesis from biodiesel and bioethanol byproducts. Carbon source composition of sugarcane bagasse hydrolysate from four-step treatment as well as culture media utilized in this study are available in Tables 1, 2, respectively. Solid media were obtained by addition of 20 g/L agar.

**Table 1:** Effect of different H_2_SO_4_ concentrations and additional treatment steps (TS) on sugarcane bagasse hydrolysate composition.

**H_**2**_SO_**4**_ (%, v/v)**		**Sugarcane bagasse hydrolysate composition (g/L)**					
		**HMF^**a**^**	**Furfural**	**Xylose**	**Arabinose**	**Acetic acid**	**Formic acid**
0.5	TS1^b^	0.001	0.002	9.32	1.27	2.47	0.03
	TS2^c^	0.002	0.002	7.78	1.03	2.37	0.39
	TS3^d^	0.003	0.003	12.92	1.68	3.60	0.06
	TS4^e^	0.000	0.002	3.15	0.54	3.02	0.12
1.0	TS1^b^	0.004	0.003	16.18	3.56	3.09	0.03
	TS2^c^	0.003	0.004	11.61	1.22	3.62	0.97
	TS3^d^	0.005	0.007	17.82	1.86	4.28	0.18
	TS4^e^	0.006	0.002	3.57	0.58	3.73	0.16
2.0	TS1^b^	0.002	0.003	18.34	1.52	3.39	0.24
	TS2^c^	0.005	0.003	16.49	1.39	6.35	2.17
	TS3^d^	0.007	0.003	24.70	2.08	3.77	1.59
	TS4^e^	0.008	0.002	4.14	0.59	4.59	0.23
3.0	TS1^b^	0.002	0.003	18.98	1.72	3.38	0.32
	TS2^c^	0.007	0.002	14.24	1.25	5.06	3.17
	TS3^d^	0.012	0.003	25.22	2.20	2.95	1.46
	TS4^e^	0.001	0.002	4.71	0.66	4.26	0.14
4.0	TS1^b^	0.003	0.003	20.58	1.81	3.68	0.50
	TS2^c^	0.000	0.003	16.12	1.04	5.10	4.00
	TS3^d^	0.000	0.003	29.32	1.90	4.99	0.49
	TS4^e^	0.001	0.002	4.62	0.64	4.78	0.17
5.0	TS1^b^	0.003	0.003	21.34	1.84	3.77	0.70
	TS2^c^	0.006	0.003	15.30	1.38	4.35	5.65
	TS3^d^	0.009	0.003	24.24	2.18	3.88	0.49
	TS4^e^	0.008	0.002	4.69	0.65	4.09	0.22

a hydroxymethylfurfural.

b Treatment Step 1–acid hydrolysis at 121°C and 1 atm for 30 min.

c Treatment Step 2–overliming with anhydrous Ca(OH)_2_.

d Treatment Step 3–heating at 80°C for 30 min under vacuum system.

e *Treatment Step 4–20% (w/v) activated charcoal at 30°C for 3 h*.

**Table 2:** Carbon energy source composition of MSM containing different biofuel byproducts for *B. glumae* MA13 cultivations in shake flasks.

**Media**	**Carbon energy source (g/L)**					
	**Arabinose**	**Fructose**	**Glucose**	**Glycerol**	**Sucrose**	**Xylose**
10 g/L sugarcane molasses	–	0.83	0.76	–	5.86	–
20 g/L sugarcane molasses	–	1.66	1.52	–	11.72	–
30 g/L sugarcane molasses	–	2.49	2.28	–	17.58	–
40 g/L sugarcane molasses	–	3.32	3.04	–	23.44	–
50 g/L sugarcane molasses	–	4.15	3.80	–	29.30	–
20 g/L sugarcane molasses plus 25% (v/v) sugarcane vinasse	–	1.72	1.60	0.30	11.72	–
20 g/L sugarcane molasses plus 50% (v/v) sugarcane vinasse	–	1.79	1.69	0.59	11.72	–
20 g/L crude glycerol	–	–	–	14.80	–	–
25% (v/v) sugarcane bagasse hydrolysate	0.47	–	–	–	–	4.45

### Bacterial Cultivations and Cell Harvest

*Burkholderia glumae* MA13 was streaked on solid LB from stock cultures for 24 h at 30°C, with subsequent transferring of bacterial colonies to 50 mL LB for 24 h at 30°C and 150 rpm. LB cultivation was submitted to serial dilutions and volumes of 0.1 mL from each dilution factor were surface plated on solid MSM containing 1 g/L (NH_4_)_2_SO_4_ as nitrogen source and added of sugarcane biorefinery related carbon sources in order to previously verify the growth capability of *B. glumae* MA13 to utilize these feedstocks. Bacterial cultivations were performed at 30°C for up to 120 h. The tested pure carbon sources were arabinose, fructose, glucose, glycerol, sucrose and xylose at a concentration of 10 g/L. Xylose was also tested added of 1, 2, and 5 g/L acetic acid or 1, 2, and 5 g/L formic acid. Among industrial byproducts, the bacterial growth was evaluated from 10 g/L sugarcane molasses; 25, 50, and 100% (v/v) sugarcane bagasse hydrolysate; 25, 50, and 100% (v/v) sugarcane vinasse; and a mixture resulting in a final concentration of 10 g/L sugarcane molasses added to 25, 50, and 100% (v/v) sugarcane vinasse. 10 g/L glycerol and 10 g/L crude glycerol were used as control for pure carbon sources and industrial byproducts, respectively.

PHA production experiments were performed in shake flasks transferring isolated bacterial colonies to 50 mL LB for 24 h, at 30°C and 150 rpm, in order to reach a minimal optical density of 0.60 at a wavelength of λ = 610 nm, which corresponds to 10^8^ cfu/mL. A volume of 5 mL from LB cultivations was used to inoculate 50 mL MSM containing the tested carbon and nitrogen sources. The first experimental set tested the PHA production from 20 g/L sucrose; 10, 20, 30, 40, and 50 g/L sugarcane molasses; 10, 20, 30, 40, and 50 g/L xylose; 25% (v/v) sugarcane bagasse hydrolysate; 25 and 50% (v/v) sugarcane vinasse added to 20 g/L sugarcane molasses. Second step of experiments evaluated the PHA production from 25 and 50% (v/v) sugarcane vinasse added to 20 g/L sugarcane molasses at different initial pHs: 5.5, 6.0, 6.5, and 7.0. In the third experimental set were observed the PHA production from MSM containing 20 g/L sugarcane molasses and 25% (v/v) sugarcane vinasse at temperatures of 27, 30, 34, 37, and 40°C, whereas the fourth step of cultivations tested the same media containing the nitrogen sources (NH_4_)_2_SO_4_, (NH_4_)_3_PO_4_, NH_4_Cl, NaNO_3_, yeast extract, autolyzed yeast or inactive dry yeast at concentrations of 1, 2 and 3 g/L and carbon/nitrogen source ratios (C/N) of 5.1, 7.7 and 15.3. Finally, *B. glumae* MA13 was cultivated in MSM containing 20 g/L glycerol and 20 g/L crude glycerol as control carbon sources. All cultivations steps were performed for 72 h at 34°C and 150 rpm, excepting the PHA production tests at different temperatures.

Bacterial cells were harvested by 10 min centrifugation at 10,600 *g* and 4°C. Supernatant was used for carbon source determination and the pellet was washed twice after two centrifugation cycles, frozen and then lyophilized for PHA analyzes. Biomass values were determined gravimetrically as grams per liter of cell dry weight (CDW). The residual biomass (X_R_) was calculated as CDW values deducted of PHA content.

### PHA Analyzes

PHA quantification and monomer composition were determined by gas chromatography coupled to mass spectrometry (GC-MS) of methyl esters (Hujberts et al., 1994). Samples of 5–10 mg lyophilized cells were subjected to methanolysis reaction and 1 μL organic phase was analyzed after split injection (1:20) by GCMS-QP2010 Ultra (Shimadzu, Japan), equipped with the column Rtx-5MS (30 m × 0.25 mm × 0.25 μm, Restek, USA). The injector temperature was 250°C and the temperature program applied to the column started at 100°C for 3 min, increasing the temperature at 8°C/min up to 210°C, which was kept for 15 min. The detector temperature was 280°C. Helium was used as carrier gas at a constant flow of 0.8 mL/min. Benzoic acid was used as internal standard. Poly(3-hydroxybutyrate-*co*-3-hydroxyvalerate) [P(3HB-*co*-3HV)] containing 14% 3HV (w/w) (Aldrich, USA) was used as external standard. The PHA content was expressed in grams per liter and the percentage (w/w) of cell dry weight (% CDW). Polymer yield (Y_PHA_) was calculated as grams of polymer per grams of consumed carbon source.

Fourier transform infrared spectroscopy (IR Prestige, Shimadzu, Japan) was performed after polymer extraction for a qualitative and comparative analysis with a P(3HB) standard (Sigma, USA). One gram lyophilized cells was added to 20 mL chloroform for PHA extraction for 48 h at 30°C and 200 rpm. Cellular debris was removed by filtration and subsequent evaporation of chloroform. The crude polymer was dissolved in a small volume of chloroform and precipitated by dropwise addition of cold ethanol. The polymer was recovered and dried overnight at 40°C for FTIR analysis. The extracted polymer was mixed with KBr and squeezed under pressure resulting in a KBr pellet. The spectra were recorded in the range 4,000–400 cm^−1^.

### Carbon Source Determination

Carbon sources were determined by HPLC apparatus equipped with UV/Vis and RID-10A detectors (Prominense, Shimadzu, Japan). Arabinose, glycerol, xylose, acetic acid and formic acid were determined using a Rezex ROA column (8% cross-linked resin hydrogen ionic form/sulfonated styrene-divinylbenzene; 300 × 7.8 mm; Phenomenex, USA). Samples of 20 μL were injected and eluted with 0.0025 M H_2_SO_4_ at a flow rate of 0.6 mL/min. The column oven temperature was kept at 65°C. Glucose, fructose and sucrose were determined by a RCM-Monosaccharide Ca^+2^ column (8% cross-linked resin calcium ionic form/sulfonated styrene-divinylbenzene; 300 × 7.8 mm; Phenomenex, USA). The injection volume was 20μL eluted with distilled water at a flow rate of 0.6 mL/min. The column oven temperature was 65°C. Furfural and hydroxymethyl furfural (HMF) were determined using a NST 18 column (C18 particle size 5 μm; 150 × 4.6 mm; NST, Brazil). Sample volumes of 10 μL were injected and eluted with a mobile phase consisted of acetonitrile/water (10:90 v/v) acidified with 1% (v/v) acetic acid at a flow rate of 0.8 mL/min. The column oven temperature was maintained at 35°C. Glucose, fructose, sucrose, arabinose, xylose, glycerol, acetic acid, formic acid, furfural, and HMF were used as external standards. The carbon sources were expressed in grams per liter. Consumed carbon source values were calculated by subtracting residual from initial carbon source after 72 h cultivation.

## Results and Discussion

### PHA Production From Sugarcane Biorefinery Byproducts

Industrial byproducts are complex carbon sources and many times they are constituted of impurities which may impair the microbial growth, such as salts and residual methanol regarding crude glycerol from biodiesel production (Cavalheiro et al., 2009) or phenolic compounds and additional contaminants from raw cane juice, when sugarcane molasses is used as a complex carbon source (Sen et al., 2019). Sugarcane vinasse is generally a low sugar, nitrogen, and phosphorus byproduct though high levels of organic matter and cations such as potassium, calcium, and magnesium are present in this low pH effluent (Christofoletti et al., 2013). Regarding sugarcane bagasse hydrolysate, many steps are required to release monomeric sugars from this lignocellulosic byproduct, which are mostly glucose and xylose, and thus make them available to microbial metabolism. However, the acid hydrolysis of sugarcane bagasse also produces toxic compounds such as furfural and HMF from sugar hydrolysis and formic and acetic acid from lignocellulose degradation that can be deleterious for microbial growth (Olsson and Hahn-Hägerdal, 1996; Silva et al., 2004; Lopes et al., 2014). Therefore*, B. glumae* MA13 was previously cultivated in solid MSM containing all tested carbon sources and different concentrations of possible growth inhibitors such as acetic and formic acids, which are obtained from sugarcane bagasse hydrolysis in significant amounts. Table 1 shows the sugar and main toxic coproducts content of sugarcane bagasse hydrolysate after a four-step pre-treatment. Acid hydrolysis from 0.5 to 5% (v/v) H_2_SO_4_ produced low amounts of furfural and HMF, whereas significative xylose content was obtained from 1 to 5% H_2_SO_4_. Although overliming is an important method to remove phenolic compounds from lignin hydrolysis (Pan et al., 2012), in this study this process resulted in a slight decrease of sugar content and an increase of acetic and formic acid concentrations. The third treatment step of sugarcane bagasse was effective to concentrate xylose and arabinose sugars and it was also a prominent treatment for removal of formic acid. Fourth pre-treatment step with activated charcoal resulted in lower amounts of sugars in the final sugarcane bagasse hydrolysate. In this sense, bacterial cultivation tests were performed using a sugarcane bagasse hydrolysate obtained after three-step pre-treatment from a low-concentration acid hydrolysis with 1% (v/v) H_2_SO_4_.

As one can see in Table 3, *B. glumae* MA13 was able to grow in MSM containing all tested pure carbon sources, excepting cultivation media added of acetic or formic acids at concentrations higher than 1 g/L. These organic acids seems to be deleterious to *B. glumae* MA13 metabolism at higher concentrations, especially acetic acid, which can be corroborated by the absence of growth in media containing 100 and 50% (v/v) sugarcane bagasse hydrolysate as sole carbon source, whose acetic acid concentrations were 4.3 and 2.1 g/L, respectively, whereas the bacterial growth was observed in MSM containing 25% (v/v) sugarcane bagasse hydrolysate with acetic acid content of 1 g/L. MSM containing 100% (v/v) sugarcane vinasse as sole carbon source or added to 10 g/L sugarcane molasses were also deleterious for bacterial growth. On the other hand, *B. glumae* was able to grow in MSM containing either 50 and 25% (v/v) pure sugarcane vinasse or added of 10 g/L sugarcane molasses. Probably, the low carbon source concentration of 100% (v/v) vinasse effluent was not impeditive for microbial growth, since it was observed bacterial growth from lower proportion volumes of this sugarcane byproduct as sole carbon source. However, the negative effect of 100% (v/v) vinasse on bacterial growth possibly can be attributed to the higher concentration of contaminants and inhibitor compounds from 100% vinasse media.

**Table 3:** Bacterial growth tests of *B. glumae* MA13 in solid MSM containing pure carbon sources and industrial byproducts for 72 h at 30°C.

**Carbon source**	**Result**
10 g/L glucose	+
10 g/L fructose	+
10 g/L sucrose	+
10 g/L sugarcane molasses	+
10 g/L glycerol	+
10 g/L crude glycerol	+
10 g/L arabinose	+
10 g/L xylose	+
10 g/L xylose plus 1 g/L acetic acid	+
10 g/L xylose plus 2 g/L acetic acid	–
10 g/L xylose plus 5 g/L acetic acid	–
10 g/L xylose plus 1 g/L formic acid	+
10 g/L xylose plus 2 g/L formic acid	–
10 g/L xylose plus 5 g/L formic acid	–
100% (v/v) sugarcane bagasse hydrolysate	–
50% (v/v) sugarcane bagasse hydrolysate	–
25% (v/v) sugarcane bagasse hydrolysate	+
100% (v/v) sugarcane vinasse	–
50% (v/v) sugarcane vinasse	+
25% (v/v) sugarcane vinasse	+
100% (v/v) sugarcane vinasse plus 10 g/L sugarcane molasses	–
50% (v/v) sugarcane vinasse plus 10 g/L sugarcane molasses	+
25% (v/v) sugarcane vinasse plus 10 g/L sugarcane molasses	+

PHA production experiments from sugarcane biorefinery related byproducts showed the promising potential of sugarcane molasses as sole carbon source or added to sugarcane vinasse as a feedstock for polymer synthesis by *B. glumae* MA13 (Table 4). Shake flask cultivations with MSM containing 10 to 50 g/L sugarcane molasses achieved polymer production values ranging from 1.3 to 2.7 g/L. Although statistical analysis (ANOVA) of the results showed that initial sugarcane molasses concentration had a significant effect (*P* < 0.05) on polymer production, intracellular polymer accumulation and polymer yield values, the concentration interval from 20 to 40 g/L sugarcane molasses was not statistically relevant regarding the polymer production with the best values ranging from 2.5 to 2.7 g/L. However, PHA production experiments with sugarcane molasses concentrations higher than 20 g/L were marked by remaining sugar content in the culture medium after 72 h, with a total sugar content (sum of glucose, fructose and sucrose concentrations) of 5.1, 9.5, and 16 g/L for MSM containing 30, 40, and 50 g/L sugarcane molasses, respectively. Due to lower sugar consumption, MSM cultivations containing 10 and 20 g/L sugarcane molasses showed the best polymer yields with 0.17 and 0.18 g/g, respectively. Intracellular polymer accumulation and CDW values obtained from sugarcane molasses were higher than those observed from pure sucrose as sole carbon source even when 25% (v/v) sugarcane vinasse was added to the culture medium, with a polymer production average value of 1.3 g/L compared to 1.1 g/L obtained from pure sucrose. The major component in molasses is sucrose whereas the glucose and fructose content of untreated sugarcane molasses are lower. Sucrose hydrolysis generally is required to improve bacteria consumption of simple sugars in molasses, and thus a pre-treatment of sugarcane molasses is necessary to release the hydrolysis products glucose and fructose prior bacterial fermentation. The sucrose hydrolysis can be accomplished via different methods using acid or alkali, enzymes such as invertases, subcritical water and cation exchange resins (Sen et al., 2019). The most classical PHA producer *Cupriavidus necator* (former *Ralstonia eutropha*) is unable to assimilate sucrose as carbon source (Arikawa et al., 2017) and a pre-treatment of sucrose-containing feedstocks is required for bacterial cultivations in order to obtain PHA biopolymers. Sen et al. (2019) reported a maximum P(3HB) production of 0.8 g/L and intracellular polymer accumulation of 27% CDW by *C. necator* from hydrothermal acid pre-treated molasses. In this work, *B. glumae* MA13 was able to produce PHAs from both sucrose and sugarcane molasses without any pre-treatment resulting in a maximum polymer production of 2.7 g/L and intracellular polymer accumulation of 46.6% CDW, which is a substantial result from a metabolic, economic, and industrial point of view.

**Table 4:** PHA production from sugarcane biorefinery related carbon sources by *B. glumae* MA13.

**Carbon source**	**Initial concentration (g/L, % v/v)**	**CDW (g/L)**	**PHA (%CDW)**	**3HB (mol%)**	**3HV (mol %)**
Sucrose	20	4.35 ± 0.19	26.53 ± 0.74	99.35	0.65
Sugarcane molasses	10	3.30 ± 0.16	41.08 ± 5.91	99.53	0.47
	20	5.80 ± 0.22	43.79 ± 5.18	99.54	0.46
	30	5.88 ± 0.30	46.61 ± 0.28	99.58	0.42
	40	5.72 ± 0.31	45.85 ± 4.98	99.45	0.55
	50	5.57 ± 0.21	39.74 ± 0.21	99.48	0.52
Sugarcane molasses plus sugarcane vinasse	20, 25%	4.46 ± 0.30	29.39 ± 2.78	99.47	0.53
	20, 50%	3.73 ± 0.18	14.07 ± 1.54	98.91	1.09
Xylose	10	0.45 ± 0.00	18.41 ± 1.19	98.87	1.13
	20	0.39 ± 0.02	18.02 ± 3.17	98.84	1.16
	30	0.43 ± 0.11	18.60 ± 0.50	98.93	1.07
	40	0.53 ± 0.07	20.46 ± 0.62	98.74	1.26
	50	0.41 ± 0.16	21.09 ± 0.76	98.51	1.49
Sugarcane bagasse hydrolysate	25%	0.61 ± 0.04	14.95 ± 2.07	98.77	1.23

Mean values of cell dry weight (CDW), PHA content as percentage of CDW besides 3HB and 3HV molar composition. Shake flask cultivations performed at 34°C and 150 rpm for 72 h, initial pH 7.0.

Despite MSM added of 25% (v/v) sugarcane bagasse hydrolysate as sole carbon source induced the polymer synthesis, PHA production values were lower than those obtained from other sugarcane biorefinery related byproducts with a PHA production average value of 0.09 g/L (additional cultivation parameters can be seen in Table 4). Lopes et al. (2014) have reported the PHA production by soil isolates in cultivation media supplemented with xylose, which resulted in CDW and intracellular polymer accumulation values at a range of 5.1–6.8 g/L and 43.2–62.5% CDW, respectively. The same authors reported a CDW value of 2.7 g/L and an intracellular polymer accumulation of 53.1% CDW from sugarcane bagasse hydrolysate by a newly isolated *Burkholderia* sp. These authors achieved such results using initial inocula of 0.5 g/L. Dilute acid hydrolysis has been usually performed in order to release the sugar content of sugarcane bagasse. However, the acid hydrolysis pre-treatment implies the formation of inhibitory coproducts that can negatively affect the bacterial growth and performance during polymer synthesis. Some authors have circumvented this backset using diluted hydrolysates and high initial cell density for microbial cultivations (Yu and Stahl, 2008; Lopes et al., 2014). On the other hand, shake flask experiments with *B. glumae* MA13 in MSM containing pure xylose as sole carbon source at concentrations ranging from 10 to 50 g/L resulted in polymer production values varying from 0.07 to 0.11 g/L, which were statistically similar to polymer production values observed from 25% (v/v) sugarcane bagasse hydrolysate, whose main carbon source was xylose and so indicating that this sugar is a poor inducer of bacterial growth and polymer synthesis by *B. glumae* MA13, since low CDW and intracellular polymer accumulation values were observed from both carbon sources.

### Influence of pH, Temperature, and Nitrogen Sources on PHA Synthesis

**Figure 1:**
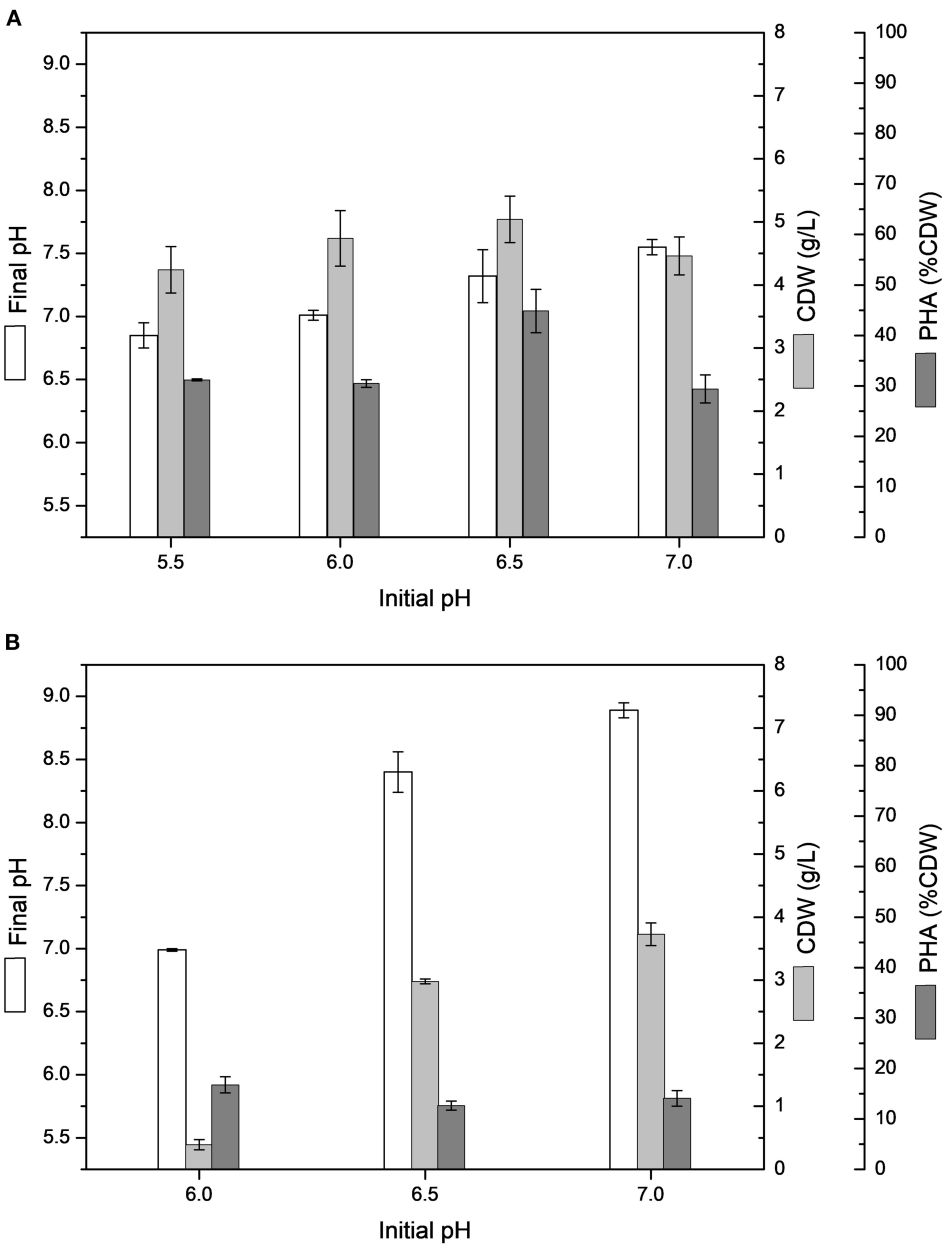
Effect of different initial pH on cell dry weight (CDW) and PHA content (% CDW) in MSM cultivations containing 20 g/L sugarcane molasses added sugarcane vinasse by *B. glumae* MA13: **(A)** 25% (v/v) sugarcane vinasse; **(B)** 50% (v/v) sugarcane vinasse. Shake flask cultivations performed at 34°C and 150 rpm for 72 h.

The PHA production experiments performed in shake flasks with MSM containing 20 g/L sugarcane molasses added of 25 or 50% (v/v) sugarcane vinasse showed higher variation between initial and final pH, especially from 50% (v/v) sugarcane vinasse, resulting in an alkaline final pH, whereas cultivations testing sugarcane molasses as sole carbon source ended with acid final pH values, varying from 6.0 to 6.4. Figure 1 shows the influence of initial pH on CDW and intracellular polymer accumulation in MSM cultivations containing 20 g/L sugarcane molasses added of 25 or 50% (v/v) sugarcane vinasse. ANOVA of the results obtained from shake flask experiments with 50% (v/v) sugarcane vinasse showed that the variation of initial pH (5.5–7.0) had a significant effect (*P* < 0.05) on CDW with increased biomass values toward to neutral initial pH (7.0), whereas the intracellular polymer accumulation values were not statistically relevant for initial pH values ranging from 6.0 to 7.0. These statistical analyzes and the absence of bacterial growth observed at initial pH 5.5 lead to assume that the increased of biomass values was determinant to increase PHA production into the pH range tested for bacterial assays. On the other hand, MSM added of sugarcane molasses and 25% (v/v) sugarcane vinasse exhibited higher CDW and intracellular polymer accumulation values than those observed from 50% (v/v) sugarcane vinasse. The initial pH 6.5 had a significant effect (*P* < 0.05) on polymer accumulation with a maximum value of 44.8% CDW and PHA production of 2.3 g/L. This polymer production is statistically similar to polymers yields observed from MSM cultivations using sugarcane molasses as sole carbon source at 34°C and pH 7.0.

**Figure 2:**
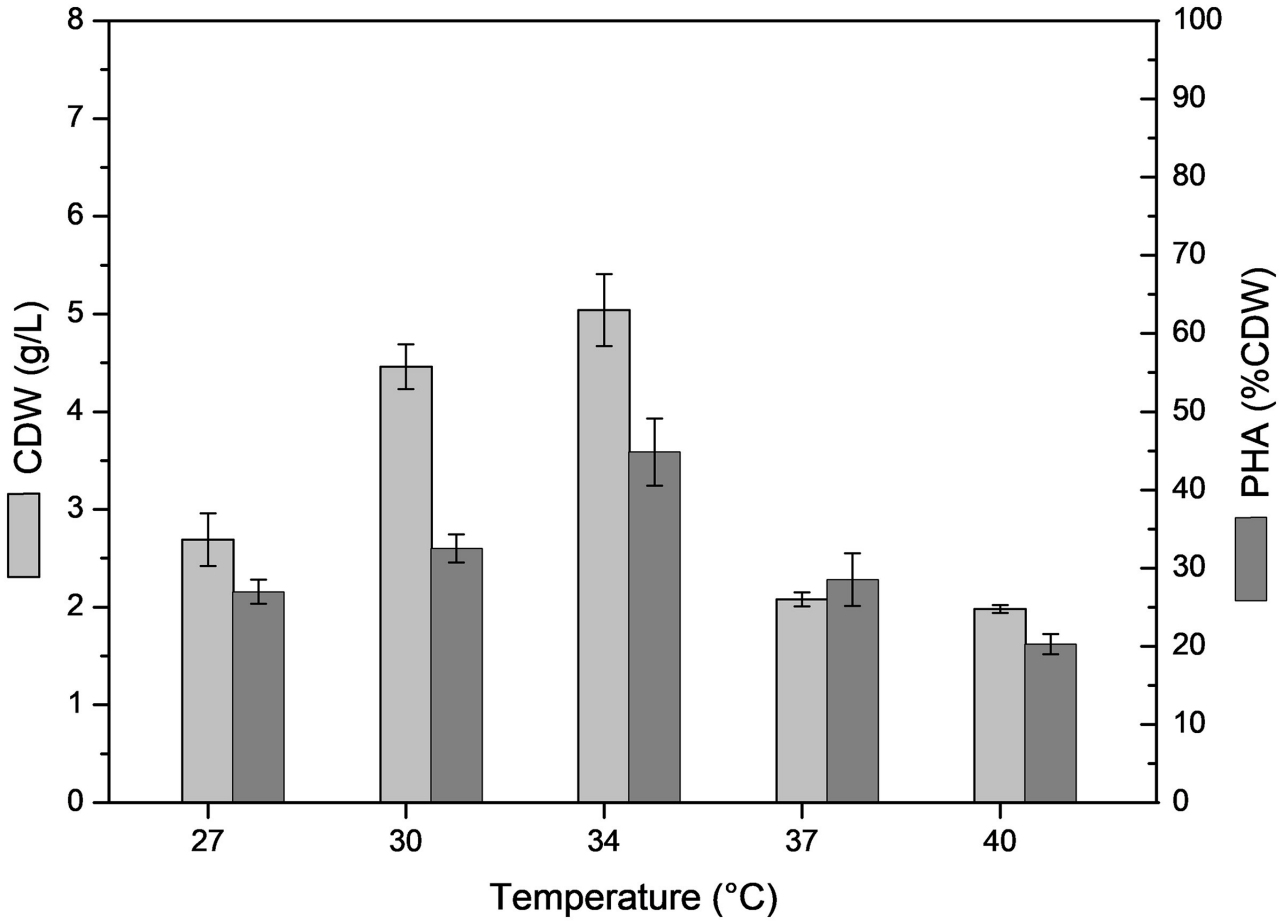
Effect of temperature on cell dry weigh (CDW) and PHA content (% CDW) in MSM cultivations containing 20 g/L sugarcane molasses plus 25% (v/v) sugarcane vinasse by *B. glumae* MA13. Shake flask cultivations performed at 150 rpm for 72 h, initial pH 6.5.

The effect of temperature on *B. glumae* MA13 growth and polymer synthesis was investigated. For this, cultivations were performed in MSM containing 20 g/L sugarcane molasses plus 25% (v/v) sugarcane vinasse at temperatures from 27 to 40°C with CDW values ranging from 2 to 5 g/L and polymer accumulation varying from 20.3 to 44.8% CDW, which resulted in the maximum polymer production of 2.7 g/L at 34°C (Figure 2). PHA-producing bacteria such as *Cupriavidus necator, Burkholderia*, and *Pseudomonas* species have showed optimum temperature of growth and polymer synthesis in the range of 28–37°C (Silva et al., 2004; Cavalheiro et al., 2009; Costa et al., 2009; Pan et al., 2012; Lopes et al., 2014) even though there are some bacterial isolates such as *Zobellella denitrificans* with an optimum temperature of 41°C (Ibrahim and Steinbüchel, 2010). Additional nitrogen sources were tested in MSM added 20 g/L sugarcane molasses and 25% (v/v) sugarcane vinasse in order to verify the bacterial performance for PHA synthesis. Even though different nitrogen sources were tested such as (NH_4_)_3_PO_4_, NH_4_Cl, NaNO_3_, yeast extract, autolyzed yeast and inactive dry yeast at concentrations of 1, 2, and 3 g/L, the best values of polymer production were obtained from 2 g/L (NH_4_)_2_SO_4_, which was equivalent a C/N ratio of 7.7 (Table 5). PHA production values from 0.2 to 2.3 g/L were observed from different nitrogen sources. Fadzil et al. (2018) reported the importance of an appropriate supply of nitrogen to achieve high cell density, from which the yeast derivative compounds such as yeast extract are among the major sources for bacterial cell growth. Their results proven a substantial improvement of cell growth from yeast extract as nitrogen source though decreased values of PHA content were observed due to a considerable increase in the biomass. In this study, *B. glumae* MA13 seems to follow this tendence from yeast derived nitrogen sources. The maximum CDW value (6.6 g/L) was obtained from 3 g/L autolyzed yeast, which was an interesting and relative low cost inducer of microbial growth. However, this nitrogen source was an ineffective substrate to trigger the polymer synthesis resulting in an intracellular polymer accumulation of 6.7% CDW after 72 h cultivation.

**Table 5:** Effect of inorganic and organic nitrogen sources on PHA production in MSM containing 20 g/L sugarcane molasses plus 25% (v/v) sugarcane vinasse by *B. glumae* MA13.

**Nitrogen source**	**C/N^**a**^**	**CDW (g/L)**	**PHA (%CDW)**	**3HB mol%**	**3HVmol %**
(NH_4_)_2_SO_4_	15.3	4.10 ± 0.57	30.72 ± 2.05	99.31	0.69
	7.7	5.04 ± 0.37	44.85 ± 4.29	99.65	0.35
	5.1	5.68 ± 0.00	29.70 ± 5.71	99.35	0.65
(NH_4_)_3_PO_4_	15.3	4.11 ± 0.12	29.11 ± 3.02	99.31	0.69
	7.7	4.11 ± 0.16	27.08 ± 1.06	99.25	0.75
	5.1	2.19 ± 0.05	25.44 ± 0.59	99.44	0.56
NH_4_Cl	15.3	4.15 ± 0.18	30.95 ± 2.03	99.34	0.66
	7.7	3.86 ± 0.80	24.77 ± 1.82	99.27	0.73
	5.1	2.80 ± 0.42	17.18 ± 0.50	99.10	0.90
NaNO_3_	15.3	3.60 ± 0.42	31.94 ± 0.24	99.49	0.51
	7.7	2.46 ± 0.12	14.00 ± 2.94	99.39	0.61
	5.1	2.15 ± 0.57	11.53 ± 0.03	99.23	0.77
Yeast extract	15.3	2.68 ± 0.39	20.51 ± 3.99	99.16	0.84
	7.7	2.79 ± 0.34	10.21 ± 3.59	98.53	1.47
	5.1	3.43 ± 0.32	8.51 ± 2.78	99.05	0.95
Autolyzed yeast	15.3	4.35 ± 0.25	14.90 ± 0.78	98.77	1.23
	7.7	4.91 ± 0.05	16.47 ± 0.19	99.50	0.50
	5.1	6.58 ± 0.11	6.72 ± 1.44	98.83	1.17
Inactive dry yeast	15.3	4.39 ± 0.19	16.93 ± 3.87	98.83	1.17
	7.7	4.21 ± 0.02	23.66 ± 2.97	98.79	1.21
	5.1	5.84 ± 0.41	3.51 ± 0.10	99.52	0.48

Mean values of cell dry weight (CDW), PHA content as percentage of CDW besides 3HB and 3HV molar composition. Shake flask cultivations performed at 34°C and 150 rpm for 72 h, initial pH 6.5.

a Carbon/Nitrogen source ratio.

### Sugarcane Molasses and Vinasse as Sustainable Feedstocks

**Figure 3:**
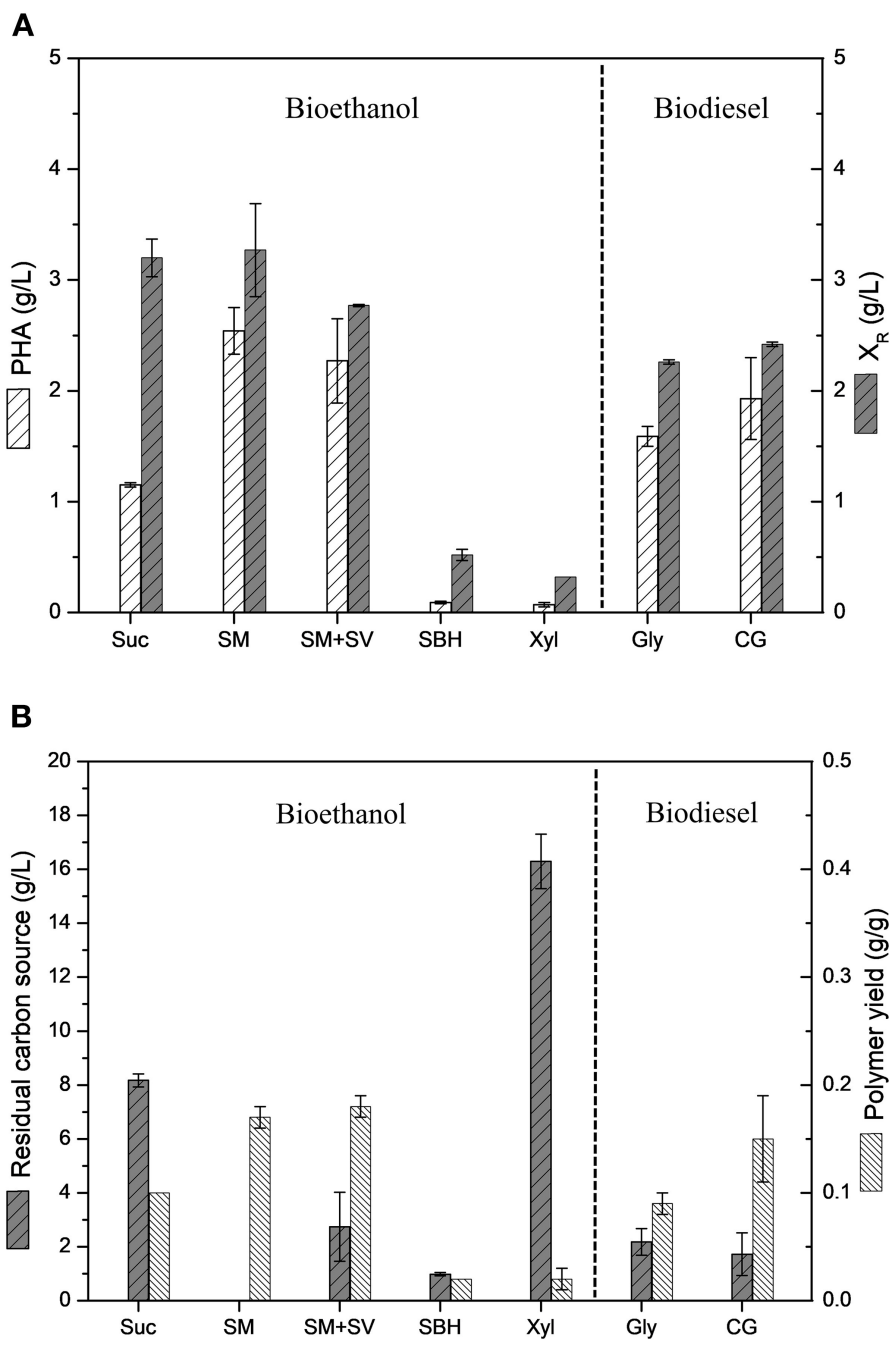
Comparative analysis of *B. glumae* MA13 performance in MSM cultivations containing different biofuel related byproducts: 20 g/L sucrose (Suc); 20 g/L sugarcane molasses (SM); 20 g/L sugarcane molasses plus 25% (v/v) sugarcane vinasse (SM+SV); 25% (v/v) sugarcane bagasse hydrolysate (SBH); 20 g/L xylose (Xyl); 20 g/L glycerol (Gly); and 20 g/L crude glycerol (CG). **(A)** Polymer production and residual biomass (X_R_); **(B)** Residual carbon source and polymer yields.

Figure 3 shows a comparative data of polymer production values and polymer yields achieved by *B. glumae* MA13 cultivations in MSM containing different biofuel related byproducts. *B. glumae* MA13 was previously isolated aiming at to obtain a bacterial strain adapted to produce PHAs using crude glycerol as sole carbon source from biodiesel plant (de Paula et al., 2019b). Fortunately, this strain seems to be adapted to additional industrial byproducts. Polymer yields of 0.17, 0.18, and 0.15 g/g were obtained from 20 g/L sugarcane molasses as sole carbon source, 20 g/L sugarcane molasses added 25% (v/v) sugarcane vinasse, and 20 g/L crude glycerol, respectively. These values correspond to 32–37% of maximum theoretical yield if considered the classical metabolic pathway of P(3HB) formation from two acetyl-CoA molecules via 3-ketothiolase, acetoacetyl-CoA reductase and PHA synthase catalysis with maximum theoretical yield values of 0.47 from glycerol, 0.48 from glucose and fructose, and 0.50 from sucrose (Gomez et al., 1996; Moralejo-Gárate, 2011). The theoretical yield assume that the carbon source is totally directed to the polymer synthesis. Since the carbon sources utilized in this work were not added to the medium in the stationary growth phase for exclusive polymer synthesis, it is reasonable to assume that part of carbon source was spent to the cell growth metabolism besides the polymer synthesis, which is known as overall polymer yield (Yamane, 1993; Gomez et al., 1996). If overall polymer yields are assumed, *B. glumae* MA13 cultivations in MSM containing sugarcane molasses plus vinasse or crude glycerol achieved conversion efficiency rates of 70–78% of carbon sources directed to PHA synthesis, which is considerably significative, since most of carbon source has been channeled to polymer synthesis. The ANOVA of the results obtained from MSM cultivations containing 20 g/L sugarcane molasses, 20 g/L sugarcane molasses added 25% (v/v) sugarcane vinasse or 20 g/L crude glycerol showed that the utilization of these different carbon sources had not a relevant effect (*P* < 0.05) on polymer yields. In other words, the bacterial performance for PHA production from the mentioned carbon sources was statistically the same regarding the polymer yields. Interestingly, the polymer yield values obtained from these byproducts were superior than those obtained from pure sources such as glycerol and sucrose.

*Burkholderia* species have become good alternatives for PHA production, which are able to consume hexose and pentose carbon sources with intracellular polymer accumulation rates reaching up to 75% CDW and in particular cases they can exhibit bacterial growth rates even faster than *C. necator*, the most well-stablished microbial model for PHA production (Li and Wilkins, 2020). Silva et al. (2004) reported the PHA production in shake flask experiments by *B. cepacia* IPT 048 and *B. sacchari* IPT 101 from sugarcane related carbon sources. The utilization of xylose as pure carbon source resulted in CDW values of 4.1 and 2.9 g/L with intracellular PHA accumulation values of 53.5 and 35% CDW, respectively. The same authors observed decreased PHA accumulation values from bacterial cultivations in shake flasks with media containing sugarcane bagasse hydrolysate, which resulted in a maximum intracellular polymer accumulation of 23.2% CDW. The PHA production from sugarcane molasses has been also reported for *Bacillus* species. Gouda et al. (2001) achieved a P(3HB) production of 0.6 g/L and an intracellular P(3HB) accumulation of 46.3% CDW from sugarcane molasses by *B. megaterium*. In this study, *B. glumae* MA13 showed similar performance to these reported polymer yields for well-known PHA-producing bacterial strains, with polymer production values ranging from 1.1 to 2.7 g/L and intracellular polymer accumulation values varying from 26.5 to 46.6% CDW from pure sugarcane molasses or added to 25% (v/v) sugarcane vinasse.

**Figure 4:**
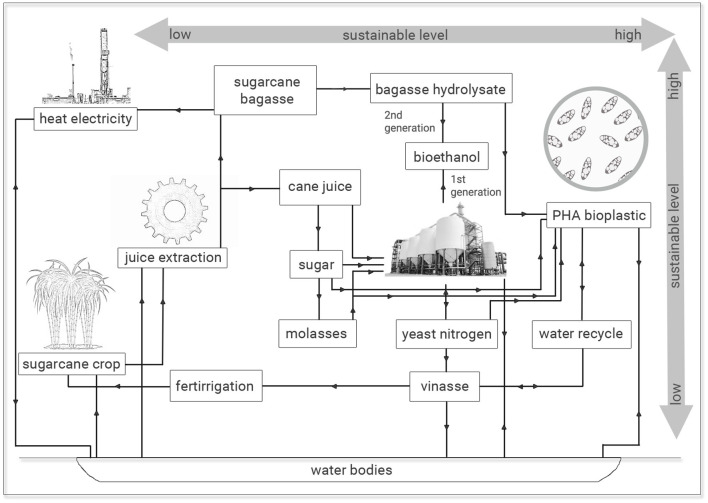
Sugarcane-to-plastic routes: from low to high sustainable processes.

The results observed are economic and ecologically attractive since similar PHA production and polymer yields were obtained from sugarcane molasses as sole carbon source or added of 25% (v/v) sugarcane vinasse. The utilization of this last residue from sugar-ethanol industry with high COD values is imperative concerning its waste management and ecosystem intake (Reis and Hu, 2017). The inadequate and indiscriminate disposal of sugarcane vinasse in soils and water bodies has implied environmental damages, since this byproduct has been especially used for fertirrigation practices with substantial changes in the physical-chemical properties of soil and consequently in its biota due to low pH and contaminants of vinasse. Genotoxic effects have been associated with vinasse besides this application to crop fields has resulted in significant increases in the GHG, especially N_2_O. If considered the discharge of vinasse in water bodies, a more catastrophic effect is expected, since vinasse is highly toxic to aquatic biota with a pollution potential hundred times higher than household sewage (Christofoletti et al., 2013). Naspolini et al. (2017) have reported the rhamnolipid production by *Pseudomonas aeruginosa* PA1 from medium containing glycerol and sugarcane vinasse. Interestingly, these authors not only obtained a biosurfactant production but also they reported a 63.2% COD removal from culture medium. Therefore, the recycle use of vinasse for microbial fermentation processes permits an integrated solution to generate value-added applications for this bioethanol byproduct and diminish the environmental impacts related to vinasse disposal in terrestrial and aquatic ecosystems. It is important to notice that vinasse is a broad contaminant and it is not exclusive of sugarcane juice fermentation. Vinasse can also be obtained from additional methods aiming at to obtain bioethanol via microbial fermentation involving beet sugar, starch crops (corn, wheat, rice and cassava) or cellulosic materials, since it is the final broth free of ethanol and microbial cells, which can be obtained at a ratio of 10–15 L per liter of ethanol (Christofoletti et al., 2013). In this study, 25% (v/v) sugarcane vinasse can be added to media containing sugarcane molasses for PHA production without conversion efficiency losses of carbon source into biopolymer with additional effluent recycle and economy of 25% (v/v) treated water for microbial cultivations (Figure 4).

### GC-MS and FTIR Analyzes

**Figure 5:**
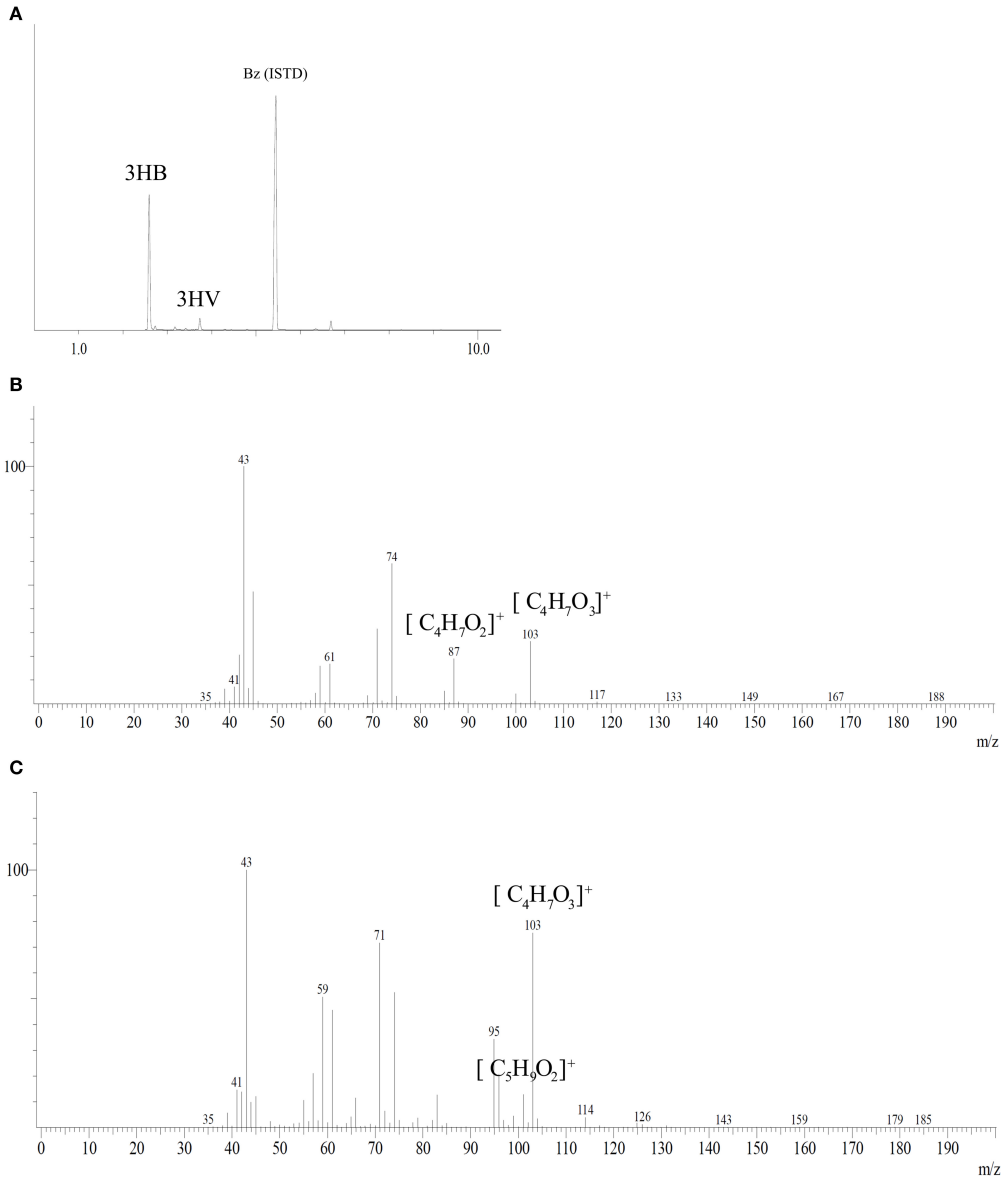
Gas chromatography coupled with mass spectrometry analysis of PHA synthesized by *B. glumae* MA13. **(A)** Peaks at retention time 2.58, 3.41, and 5.45 min were identified as 3HB, 3HV, and benzoic acid internal standard, respectively. Mass spectra of electron ionized methyl-esters of 3-hydroxyalkanoates: **(B)** 3HB; **(C)** 3HV.

PHA analysis after methanolysis reaction showed a polymer mainly constituted of 3HB monomer with low fractions of 3HV from tested carbon sources with a maximum of 1.5 mol%. Figure 5 shows the chromatogram and mass spectra of the polymer produced by *B. glumae* MA13 from MSM containing 20 g/L glycerol and 25% (v/v) sugarcane vinasse after 72 h cultivation at 34°C and 150 rpm. The peaks at 2.58, 3.41, and 5.45 min were identified as 3HB, 3HV and benzoic acid internal standard, respectively, according to the mass spectra of methyl esters of 3-hydroxyalkanoates. The fragment m/z 103 is characteristic of methyl esters formed by α-cleavage of the hydroxyl functional group, whereas the monomers 3HB and 3HV can be identified by analysis of the fragment of m/z [M-31] (loss of methoxyl group from the methyl ester), which is the difference between the total molecule and the assigned ion and results in the fragment m/z 87 for 3HB and the fragment m/z 101 for 3HV monomer (Lee and Choi, 1995).

**Figure 6:**
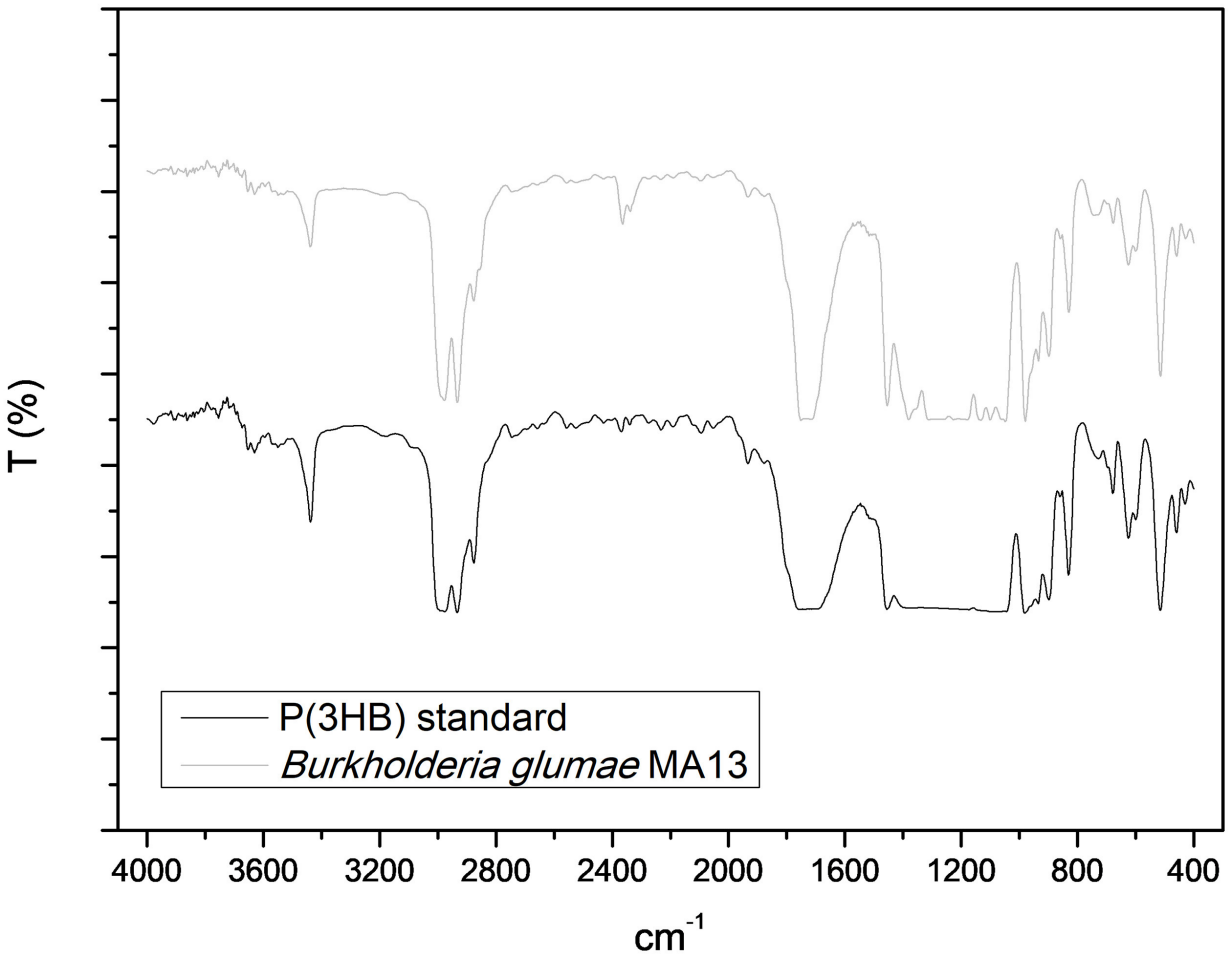
Fourier transformed infrared spectroscopy analysis of P(3HB) standard and the chloroform extracted PHA synthesized by *B. glumae* MA13.

Figure 6 shows the IR spectra of P(3HB) standard and the PHA produced by *B. glumae* MA13, which was mainly constituted of P(3HB). The peak absorption band of 1,732 cm^−1^ from P(3HB) standard and 1,730 cm^−1^ from *B. glumae* MA13 extracted polymer corresponds to the ester carbonyl group (C=O) of P(3HB) polymer. The peaks at 2,933 and 2,931 cm^−1^ from standard and extracted PHA refer to as C–H stretching vibration of methyl and methylene. The peak 1,455 cm^−1^ is characteristic of asymmetric stretching of –CH_3_ and –CH_2_ groups. At 1,385 cm^−1^ is observed the symmetric deformation of –CH_3_ and –CH_2_ groups. Finally, the absorption peaks from 1,200 to 900 cm^−1^ are related to the vibration of C–O–C bonds (Xiao and Jiao, 2011).

## Conclusion

*Burkholderia glumae* MA13, an originally isolated bacterial strain aiming at the production of PHAs utilizing crude glycerol from biodiesel industry, was tested to synthesize these biopolymers from different feedstocks related to sugarcane biorefineries resulting in high intracellular polymer accumulation rates, especially from non-pre-treated industrial byproducts, with highlights to sugarcane molasses either as sole carbon source or added of vinasse achieving the same polymer yields. The utilization of sugarcane vinasse in the culture medium offered ecological benefits to save water and due to a fermentative recycle of this industrial effluent besides the additional economic advantage to generate value-added chemicals via fermentation process such as highly biodegradable plastics. Therefore, *B. glumae* MA13 showed to be a promising bacterial strain in order to synthesize PHAs into a sustainable sugarcane biorefinery concept using low cost feedstocks totally integrated into a productive chain from sugarcane milling to the disposal of bioethanol effluents.

## Data Availability Statement

The original contributions presented in the study are included in the article/supplementary materials, further inquiries can be directed to the corresponding author/s.

## Author Contributions

CP, FP-E, and JC conceived the study. CP performed the experiments with collaboration of MR, LC, and NO. CP, FP-E, and AA contributed to design and organized the database. JC obtained funding to financially support this study. CP and FP-E wrote the manuscript in consultation with JC. All authors read and approved the final manuscript.

## Conflict of Interest

The authors declare that the research was conducted in the absence of any commercial or financial relationships that could be construed as a potential conflict of interest.
